# Analysis of Periostin, TGF-β, and SLUG Expression in Inflammatory Bowel Disease in Pediatric Patients and Their Clinical Implications

**DOI:** 10.3390/jcm15020845

**Published:** 2026-01-20

**Authors:** Patrycja Sputa-Grzegrzolka, Anna Socha-Banasiak, Aleksandra Piotrowska, Mateusz Olbromski, Monika Mrozowska, Aneta Popiel-Kopaczyk, Aleksandra Gurzkowska, Krzysztof Paczes, Elzbieta Czkwianianc, Hanna Romanowicz, Piotr Dziegiel, Bartosz Kempisty

**Affiliations:** 1 Division of Anatomy, Department of Human Morphology and Embryology, Faculty of Medicine, Wroclaw Medical University, 50-368 Wroclaw, Poland; 2 Department of Gastroenterology, Allergology and Pediatrics, Polish Mother’s Memorial Hospital-Research Institute, 93-338 Lodz, Poland; 3 Division of Histology and Embryology, Department of Human Morphology and Embryology, Faculty of Medicine, Wroclaw Medical University, 50-368 Wroclaw, Poland; 4 Laboratory of Cancer Genetics, Department of Pathology, Polish Mother’s Memorial Hospital Research Institute, 93-338 Lodz, Poland; 5 Physiology Graduate Faculty, North Carolina State University, Raleigh, NC 27607, USA; 6 Department of Veterinary Surgery, Institute of Veterinary Medicine, Nicolaus Copernicus University, 87-100 Torun, Poland; 7 Department of Stem Cells and Regenerative Medicine, Institute of Natural Fibres and Medicinal Plants, 60-630 Poznan, Poland

**Keywords:** IBD, Crohn’s disease, ulcerative colitis, intestinal wall fibrosis, periostin, SLUG, TGF-β, EMT, pIBD

## Abstract

**Background:** Pediatric inflammatory bowel disease (pIBD), including Crohn’s disease (CD) and ulcerative colitis (UC), is characterized by chronic intestinal inflammation and fibrosis. Identifying molecular mediators involved in inflammation and tissue repair is critical for improving disease management. **Objective:** To examine the expression of periostin, TGF-β, and SLUG in pIBD and assess their potential roles in intestinal inflammation, fibrosis, and mucosal healing. **Methods:** Intestinal biopsies from 33 pediatric patients (11 CD, 22 UC) and 10 healthy controls were analyzed immunohistochemically. Quantitative PCR evaluated *POSTN*, *TGF-β1*, and *SNAI2* expression in 22 patients and 6 controls. Correlations with fecal calprotectin, the Pediatric Crohn’s Disease Activity Index (PCDAI), and the Pediatric Ulcerative Colitis Activity Index (PUCAI) were determined. **Results:** Periostin, TGF-β, and SLUG expression were significantly increased in pIBD compared with controls. Periostin levels were higher in CD than in UC. All markers correlated positively at mRNA and protein levels. Notably, periostin showed an inverse correlation with fecal calprotectin and PCDAI scores. **Conclusions:** Periostin, TGF-β, and SLUG may represent biomarkers of pIBD activity. Periostin appears to mediate inflammation and promote mucosal fibrosis or repair, and its inverse association with disease activity suggests a potential therapeutic role in pIBD.

## 1. Introduction

Inflammatory bowel disease (IBD) is a group of chronic gastrointestinal disorders characterized by periods of exacerbation and remission that include Crohn’s disease (CD), ulcerative colitis (UC), and IBD-unclassified (IBD-U) [1]. The incidence of IBD, particularly CD and UC, is steadily increasing in both children and adults worldwide [2,3,4,5,6].

IBD is often diagnosed at a young age, and in pediatric patients four subgroups are distinguished based on age at onset: adolescent-onset (10–18 years), early-onset (6–9 years), very early-onset (VEO-IBD, <6 years), and infantile-onset (<2 years) [7,8,9]. Pediatric IBD (pIBD) generally exhibits faster progression, greater extent of intestinal involvement, and more complex phenotypes compared to adult-onset IBD [1,10,11,12]. The disease is driven by genetic, microbial, immunological, and environmental factors, along with their interactions [2,13,14,15]. A positive family history is also a major risk factor in pIBD [8].

The clinical presentation of IBD depends on the location and extent of lesions [2,13]. In CD, inflammation most often affects the ileocolonic region, though it may occur throughout the gastrointestinal tract [8,16]. Transmural inflammation leads to destruction of the intestinal wall, fibrosis, and formation of strictures and fistulas [6,17,18]. UC, in contrast, involves continuous superficial inflammation spreading proximally from the rectum [8,19]. Three forms are distinguished—proctitis, left-sided colitis, and pancolitis—depending on disease extent. Clinically, UC patients experience rectal bleeding, tenesmus, and urgency [8,19]. Disease activity is typically assessed using fecal calprotectin and pediatric scoring systems: the Pediatric Crohn’s Disease Activity Index (PCDAI) and the Pediatric Ulcerative Colitis Activity Index (PUCAI) [20,21]. Calprotectin, a calcium-binding protein constituting nearly 45% of neutrophil cytoplasmic content, reflects the severity of mucosal inflammation [22].

Fibrosis is a frequent consequence of chronic intestinal inflammation [7,23,24]. Approximately 30–50% of CD and 1–12% of UC patients develop fibrosis-related complications [25,26,27,28], which negatively affect disease course and quality of life [29,30]. Strictures are classified as inflammatory (potentially reversible, responsive to anti-TNF-α therapy) or fibrotic (requiring endoscopic or surgical intervention) [31,32]. In CD, fibrotic strictures are often irreversible and recur even after resection [24,33]. Disease progression can follow non-stricturing (B1), penetrating (B2), or stricturing (B3) behavior [34,35], with most patients eventually developing structural complications [36,37,38,39]. In UC, fibrosis is typically limited to the mucosa and submucosa, although severe cases may exhibit deeper wall involvement and impaired motility [25,40,41,42].

A key mechanism underlying tissue remodeling in chronic inflammation is epithelial–mesenchymal transition (EMT), during which epithelial cells lose polarity and adhesion and acquire mesenchymal characteristics such as motility, invasiveness, and extracellular matrix (ECM) production [43,44]. EMT is regulated by transcription factors including SNAIL, SLUG, TWIST, ZEB1, and ZEB2, which repress epithelial markers (e.g., E-cadherin) while promoting mesenchymal proteins (e.g., vimentin, N-cadherin). This process is modulated by TGF-β, Wnt, Notch, and Hedgehog signaling and can be induced by hypoxia and inflammatory cytokines [45,46].

In IBD, overexpression of EMT-related transcription factors such as SNAIL and SLUG has been observed in damaged intestinal crypts, extending to mesothelial and subserosal fibroblasts, particularly in fibrotic areas [47]. Increased TGF-β1 expression and nuclear accumulation of SLUG in fibrotic regions have been reported, accompanied by β-catenin activation and fibroblast proliferation [48], while TGF-β1 was shown to induce SLUG-dependent L1CAM upregulation and epithelial motility [49]. In Crohn’s disease-associated fistulae, SLUG expression is predominantly localized to peripheral fibrotic regions, suggesting its involvement in stromal remodeling [50].

Periostin, an extracellular matrix protein encoded by the *POSTN* gene, regulates cell–matrix adhesion, proliferation, and differentiation through interactions with integrins [51,52,53]. Initially identified in bone tissue [54,55], periostin is now known to participate in diverse physiological and pathological processes including skin and cardiac remodeling [51,56,57,58,59], bronchial fibrosis in asthma [60], and bone marrow fibrosis [61]. Recent studies suggest that periostin may also contribute to intestinal fibrosis in IBD [62,63].

Periostin expression is often linked with EMT-related transcription factors, particularly SLUG, in fibrotic and neoplastic tissues. For example, periostin enhances EMT through the ILK–Akt–SLUG pathway in endometrial epithelial cells [64], while co-expression of periostin and SLUG has been observed in nasopharyngeal carcinoma [65] and in epicardium-derived cells during cardiac fibrosis [66]. Importantly, periostin is recognized as a key mediator of inflammatory and fibrotic remodeling across various organs [53], suggesting it may play a similar role in IBD.

Considering these findings, our study aimed to analyze the expression of periostin, SLUG, and TGF-β in the intestinal tissues of pediatric IBD patients and to assess potential correlations between these molecular markers and clinical disease activity.

## 2. Materials and Methods

### 2.1. Materials

The material for the experiments consisted of collected and archived tissue biopsy fragments from the mucosa of the ileum and colon, obtained during colonoscopy, fixed in formalin, and then preserved in paraffin blocks. Additionally, the biopsy specimens were preserved in RNAlater solution for later molecular analyses.

In our study, we analyzed the immunohistochemical expression of periostin, TGF-β, and SLUG in 33 pediatric patients hospitalized at the Department of Gastroenterology, Allergology, and Pediatrics at the Polish Mother’s Health Center Institute in Łódź with diagnosed IBD, including 11 Crohn’s disease (CD) and 22 ulcerative colitis (UC) cases. Diagnosis of IBD, CD, or UC was based on clinical, endoscopic, and histopathological criteria, and disease activity was assessed using the PCDAI (for CD) and PUCAI (for UC) scales. All biopsies were collected during active disease. The control group consisted of 10 children without IBD in whom organic gastrointestinal disease was excluded. These children were hospitalized for abdominal pain and/or chronic diarrhea, but during colonoscopy no macroscopic abnormalities were observed, and histological evaluation of biopsies from the cecum, transverse colon, sigmoid colon, or rectum showed no features of IBD. Inclusion criteria for patients with IBD included newly diagnosed disease or clinical features of exacerbation, while exclusion criteria for all participants included: acute conditions (trauma, infection, exacerbation of chronic disease), chronic inflammatory diseases, chronic kidney diseases, and endocrine disorders. Biopsies from representative sites (terminal ileum, cecum, transverse colon, and sigmoid colon or rectum) were collected for immunohistochemical and molecular analyses. In some cases, biopsies from sections showing the most severe macroscopic changes were used. For molecular testing by real-time PCR, biopsies from 22 pIBD patients and 6 controls were used.

**Figure 1:**
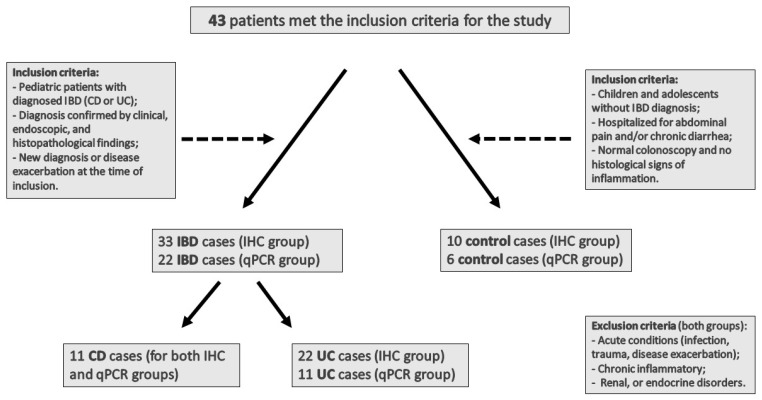
Study group composition and inclusion/exclusion criteria. A total of 43 pediatric patients met the inclusion criteria for the study. The study group included 33 IBD cases (22 UC and 11 CD) analyzed by immunohistochemistry (IHC) and 22 IBD cases (11 UC and 11 CD) analyzed by quantitative PCR (qPCR). The control group comprised 10 cases in the IHC group and 6 cases in the qPCR group.

Demographic and clinical data of participants, including age, sex, and relevant clinical parameters, are summarized in Table 1 and Table 2. No demographic or other clinical factors were used to determine eligibility for participation in the study. The study was approved by the Ethics Committee at the Polish Mother’s Health Center Institute, No. 79/2019, dated 18 June 2019. All parents and children ≥ 16 years old provided written informed consent prior to participation. Detailed information regarding patient numbers, group composition, and inclusion/exclusion criteria is presented in Figure 1.

### 2.2. Immunohistochemistry (IHC)

IHC were performed on 4 µm thick paraffin sections using Autostainer Link48 (Agilent, Santa Clara, CA, USA). Deparaffinization, rehydration, and epitope retrieval procedures were performed in EnVision FLEX Target Retrieval Solution (Agilent) (97 °C, 20 min; pH 9) using PTLink (Agilent). Activity of endogenous peroxidase was blocked using EnVision FLEX Peroxidase-Blocking Reagent (5 min; RT, Agilent). The following primary antibodies were applied for 20 min at RT: periostin (1:100, NBP1–82472, Novus Biologicals, Littleton, CO, USA), TGF-β1 (1:100, NBP2–22114, Novus Biologicals), and SLUG (1:50, sc-166476, Santa Cruz Biotechnology, Dallas, TX, USA). Next, the sections were incubated with secondary antibodies conjugated with horseradish peroxidase (EnVision FLEX/HRP, 20 min at RT) (Agilent). The reactions were visualized using freshly prepared DAB (diaminobenzidine), with incubation for 10 min at RT. Additionally, all the slides were counterstained with FLEX Hematoxylin (Agilent) for 5 min at RT and dehydrated in graded ethanol concentrations (70%, 96%, 99.8%) and then xylene. Finally, the slides were mounted in Mounting Medium (Agilent). All antibodies were diluted in Antibody Diluent (Agilent). Positive controls for the antibodies were performed using tissues recommended by the manufacturers, according to the provided protocols: breast carcinoma for anti-periostin and anti-SLUG antibodies, and lymph node for the anti-TGF-β antibody. The negative control consisted of reactions performed without primary antibodies.

### 2.3. RNA Isolation, cDNA Synthesis, and qPCR

Total RNA was extracted using the RNeasy Mini Kit (Qiagen, Hilden, Germany), following the manufacturer’s instructions. RNA was reverse-transcribed into cDNA using the High Capacity cDNA Reverse Transcription Kit (Applied Biosystems, Foster City, CA, USA). Quantitative real-time PCR (RT-qPCR) reactions were performed in 20 µL volumes using the TaqMan Universal PCR Master Mix (Applied Biosystems) and run on a 7900HT Fast Real-Time PCR System (Applied Biosystems). The following TaqMan Gene Expression Assays were used: *TGF-β1*–Hs07289533_m1, *POSTN*–Hs01566750_m1, *SNAI2* (SLUG)–Hs00161904_m1, and *ACTB*–Hs99999903_m1 as a reference gene. Thermal cycling conditions were 50 °C for 2 min (polymerase activation), 94 °C for 10 min (initial denaturation), followed by 40 cycles of 94 °C for 15 s (denaturation) and 60 °C for 1 min (annealing/extension). Each reaction was performed in triplicate. Relative gene expression was calculated using the 2^−ΔΔCT^ method, normalized to ACTB expression. Water was used as a no-template control (NTC) for the selected primers in the RT-PCR reactions to confirm the absence of contamination and non-specific amplification.

### 2.4. Histological Analysis

Positive cytoplasmic IHC reactions for periostin, SLUG, and TGF-β antigens were evaluated using the immunoreactive score (IRS) proposed by Remmele and Stegner [67]. This method takes into account two parameters: the percentage of positively stained cells (A) and the staining intensity (B). The final score was calculated as the product of these two values (A × B). Detailed criteria are presented in Table 3. According to the IRS scale, a reaction was considered positive when at least 1% of the cells in the specimen exhibited weak staining. In addition, the nuclear expression of the SLUG protein was assessed using a semi-quantitative scoring system based on the proportion of tumor cells showing positive nuclear staining: 0%—0 points; 1–10%—1 point; 11–25%—2 points; 26–50%—3 points; and 51–100%—4 points. All specimens were examined with an OLYMPUS BX-41 light microscope (Olympus, Tokyo, Japan) by two independent pathologists. In cases of discrepant results, the slides were re-evaluated jointly until a consensus was achieved. Inter-observer agreement prior to consultation was evaluated using the intraclass correlation coefficient (ICC) with a two-way random-effects model, absolute agreement, and single measurements. The obtained ICC value was 0.95, indicating excellent agreement between observers. In accordance with the antibody manufacturers’ recommendations, positive and negative control reactions were performed prior to the main IHC analyses.

### 2.5. Statistical Analysis

The collected results were subjected to statistical analysis using GraphPad Prism 5.0 (La Jolla, CA, USA) and STATISTICA 13.3 (StatSoft Inc., Tulsa, OK, USA).

The Kolmogorov–Smirnov test was used to assess the normality of the data distribution. Depending on the data characteristics, parametric tests (Student’s *t*-test or one-way ANOVA) were used for normally distributed variables, whereas nonparametric tests (Mann–Whitney U or Kruskal–Wallis with Dunn’s post hoc test) were applied for non-normally distributed data or unequal group distributions. Following a significant Kruskal–Wallis test, multiple pairwise comparisons were performed using Dunn’s post hoc test with Bonferroni–Dunn correction for multiple testing. Correlations between variables were evaluated using Pearson’s or Spearman’s correlation coefficients, depending on data distribution.

In addition, an a priori power analysis was performed using STATISTICA 13 to determine the minimum sample size required to detect effects of large magnitude (as reported in previous studies). For the planned comparisons (two-tailed *t*-test), power analysis was conducted assuming α = 0.05, power (1–β) = 0.80, and an expected strong effect size (Cohen’s d = 0.8). The analysis indicated that a minimum of N = 58 participants would be required to achieve sufficient statistical power (39 vs. 19). Due to recruitment limitations, the final sample included 43 participants (33 vs. 10).

A post hoc power analysis was conducted in Statistica 13 using the classical analytical method based on the statistical distributions of the applied tests (t, F, or χ^2^). The analysis assumed α = 0.05 and a moderate effect size (Cohen’s d = 0.6). The actual sample sizes for each comparison were used in the calculations. This approach was chosen to estimate the achieved statistical power and to assess whether the study had sufficient sensitivity to detect effects of moderate magnitude.

All results were considered statistically significant at *p* < 0.05.

## 3. Results

### 3.1. Analysis of Periostin Immunihsitochemical (IHC) Expression

**Figure 2:**
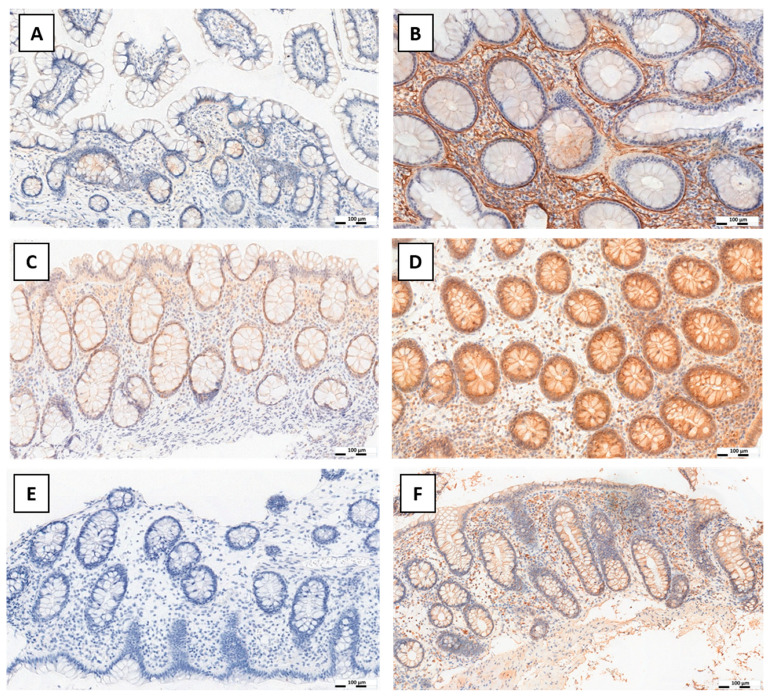
Tissue fragments from intestinal mucosa biopsies showing expression by IHC of periostin in the extracellular matrix of the lamina propria of the mucosa and submucosa of control (weak, (**A**)) and pIBD cases ((**B**), strong), nuclear and cytoplasmic expression of SLUG in epithelial cells and in connective tissue cells of the lamina propria of the mucosa and submucosa of control (weak, (**C**)) and pIBD cases (strong, (**D**)), and cytoplasmic expression of TGF-β in epithelial cells and in connective tissue cells of the lamina propria of the mucosa and submucosa of control (lack of expression, (**E**)) and pIBD cases (strong, (**F**)).

Diverse cytoplasmic expression immunoreactive score (IRS) of periostin was demonstrated in connective tissue ECM and cells of the submucosa and lamina propria of the intestinal mucosa (stroma) (Figure 2A,B).

Positive expression was demonstrated in all cases in the study group and in 9/10 cases in the control group (Table 1).

**Figure 3:**
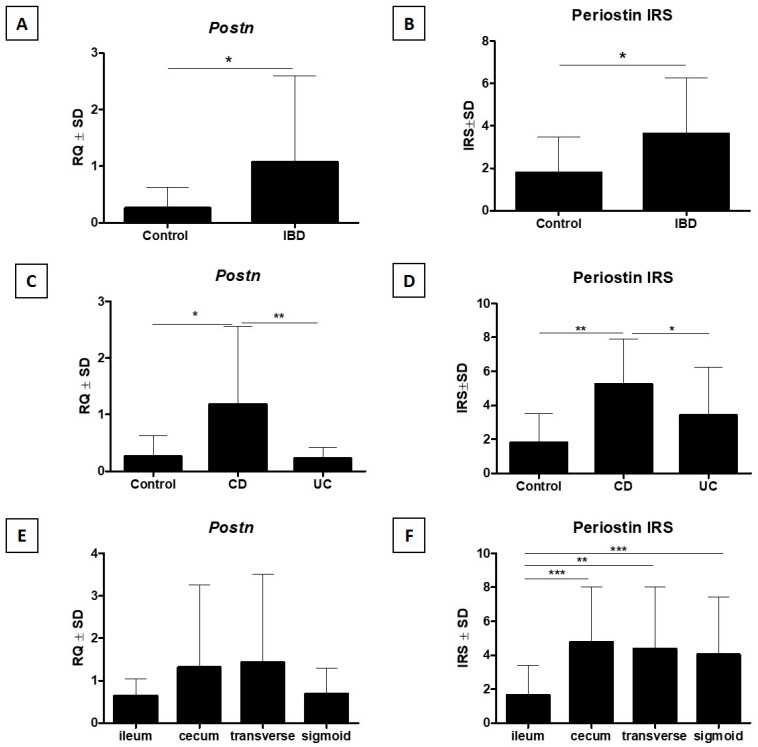
Significantly higher expression of *POSTN* mRNA (**A**) and periostin expression by IHC (**B**) in the IBD group compared with the control group. A more detailed analysis showed higher expression of *POSTN* mRNA (**C**) and periostin protein by IHC (**D**) in the CD patient group compared with UC and control. Additionally, *POSTN* mRNA expression (trend) (**E**) and periostin expression by IHC (**F**) were higher in the ileum compared with all large-intestine segments (respectively: Mann–Whitney U test (**A**,**B**), Kruskal–Wallis test with Dunn’s post hoc test (**C**–**F**), * *p* < 0.05, ** *p* < 0.01, *** *p* < 0.001). IBD—inflammatory bowel disease; CD—Crohn’s disease; UC—ulcerative colitis; IRS—immunoreactive score; *POSTN*—periostin.

Statistical analysis of the results showed a significantly higher periostin IRS by IHC in IBD cases compared to the control group (*p* < 0.05, Mann–Whitney U test, Figure 3B). In addition, significantly higher periostin IRS by IHC was demonstrated in the group of patients with CD compared to the control group and compared to patients with UC. A trend towards higher expression of periostin was also observed in the group of patients with UC compared to the control group (*p* < 0.01, Kruskal–Wallis test with Dunn post hoc test, Figure 3D). A comparative analysis of periostin IRS by IHC in individual sections of the intestine showed a significantly higher expression of this marker in all sections of the large intestine (cecum, transverse colon, and sigmoid colon or rectum) compared to the terminal ileum (*p* < 0.001, Kruskal–Wallis test, Figure 3F).

### 3.2. Analysis of POSTN mRNA Expression

Statistical analysis of *POSTN* mRNA expression showed significantly higher expression of this marker in IBD cases compared to the control group (*p* < 0.05, Mann–Whitney U test, Figure 3A). In addition, significantly higher *POSTN* expression was demonstrated in the group of patients with CD compared to the control group and compared to UC patients, but no difference was found between *POSTN* expression in the control group and UC patients (*p* < 0.01, Kruskal–Wallis test with Dunn post hoc test Figure 3C). A comparative analysis of *POSTN* expression in individual sections of the intestine showed a statistically insignificant trend toward higher expression of this marker in all sections of the large intestine (cecum, transverse colon, and sigmoid colon or rectum) compared to the terminal ileum (*p* > 0.05, Kruskal–Wallis test, Figure 3E). Detailed data regarding *POSTN* mRNA expression are presented in Table 2.

### 3.3. Analysis of SLUG Expression

Diverse cytoplasmic-nuclear expression of SLUG was demonstrated in both intestinal epithelial cells and connective tissue cells of the submucosa and lamina propria (stroma) (Figure 2C,D). Detailed data on IHC expression of SLUG are provided in Table 1.

#### 3.3.1. Analysis of Cytoplasmic Expression of SLUG in Epithelial Cells

**Figure 4:**
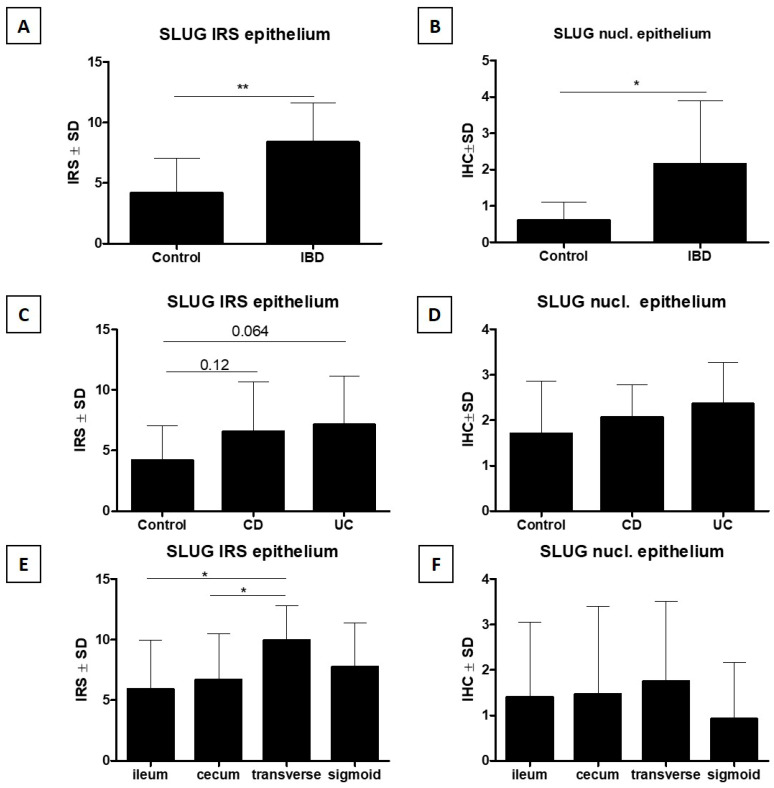
In intestinal epithelial cells, significantly higher SLUG expression by IHC was observed in the IBD group compared with the control group, both in the cytoplasm (**A**) and in the cell nucleus (**B**). A more detailed analysis showed higher cytoplasmic SLUG expression (**C**) in the CD and UC patient groups compared with the control group (a trend bordering on statistical significance). A similar but statistically insignificant trend was observed for nuclear SLUG expression. In addition, cytoplasmic SLUG expression was significantly higher in the transverse colon compared with the ileum and cecum (**E**), which was not observed for nuclear SLUG expression (**F**) (respectively: Mann–Whitney U test (**A**,**B**), Kruskal–Wallis test with Dunn’s post hoc test (**C**–**F**), * *p* < 0.05, ** *p* < 0.01). IBD—inflammatory bowel disease; CD—Crohn’s disease; UC—ulcerative colitis; SLUG—SNAIL family transcriptional repressor 2; IRS—immunoreactive score; nucl.—nuclear.

Statistical analysis of the results showed a significantly higher cytoplasmic IHC expression of SLUG in epithelial cells (epithelial SLUG IRS) in IBD cases compared to the control group (*p* < 0.01, Mann–Whitney U test, Figure 4A). In addition, a statistically insignificant trend toward higher epithelial SLUG IRS by IHC in epithelial cells was demonstrated in the group of patients with CD and UC compared to the control group (*p* > 0.05, Kruskal–Wallis test with Dunn post hoc test, Figure 4C). A comparative analysis of epithelial SLUG IRS by IHC in epithelial cells in individual sections of the intestine showed a significantly higher expression of this marker in the transverse colon compared to the cecum and ileum, and a statistically insignificant trend toward higher epithelial SLUG IRS by IHC in epithelial cells in the sigmoid colon or rectum compared to the ileum and cecum (*p* > 0.05, Kruskal–Wallis test with Dunn post hoc test, Figure 4E).

#### 3.3.2. Analysis of Nuclear Expression of SLUG in Epithelial Cells

Statistical analysis of the results showed a significantly higher nuclear IHC expression of SLUG (epithelial nuclear SLUG) in epithelial cells in IBD cases compared to the control group (*p* < 0.05, Mann–Whitney U test, Figure 4B). In addition, a statistically insignificant trend toward higher epithelial nuclear SLUG expression by IHC in epithelial cells was demonstrated in the group of patients with CD compared to the group of patients with UC, as well as to the control group (*p* > 0.05, Kruskal–Wallis test with Dunn post hoc test, Figure 4D). A comparative analysis of epithelial nuclear SLUG expression by IHC in epithelial cells in individual sections of the intestine did not reveal any significant differences between these sections (*p* > 0.05, Kruskal–Wallis test with Dunn post hoc test, Figure 4F).

#### 3.3.3. Analysis of Cytoplasmic Expression of SLUG in the Connective Tissue of the Lamina Propria of the Mucosa and Submucosa

**Figure 5:**
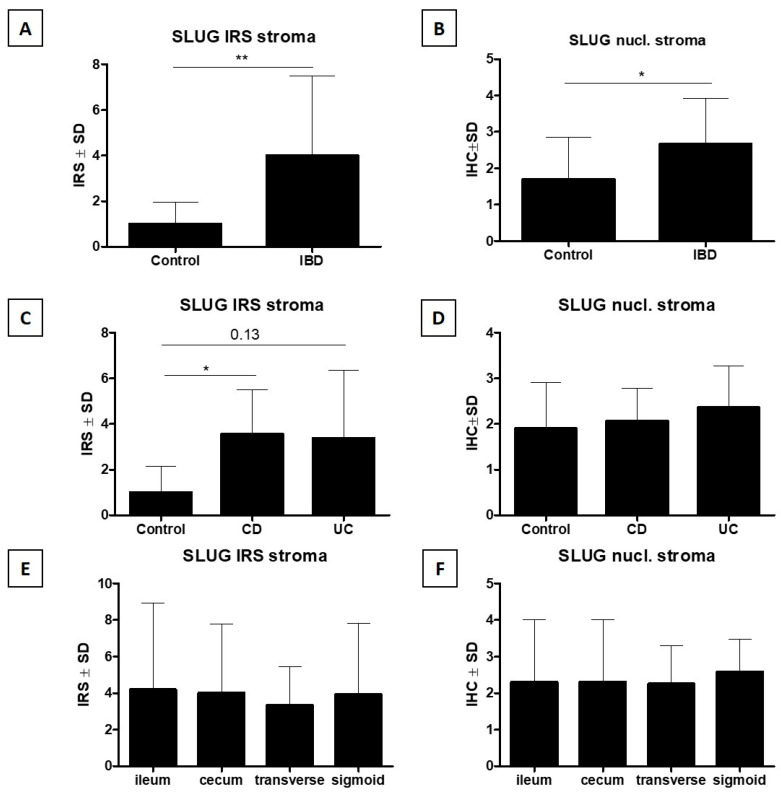
In the connective tissue cells of the intestinal submucosa, significantly higher SLUG expression by IHC was observed in the IBD group compared with the control group, both in the cytoplasm (**A**) and in the cell nucleus (**B**). A more detailed analysis showed higher cytoplasmic SLUG expression (**C**) in the CD and UC patient groups compared with the control group (in the case of UC, the trend was borderline statistically significant). Similarly, for nuclear SLUG expression there was a statistically insignificant trend toward higher expression in CD and UC compared with the control group. Additionally, for nuclear and cytoplasmic SLUG expression (**E**,**F**), no significant differences were observed in the examined intestinal segments (respectively: Mann–Whitney U test (**A**,**B**), Kruskal–Wallis test with Dunn’s post hoc test (**C**–**F**), * *p* < 0.05, ** *p* < 0.01). IBD—inflammatory bowel disease; CD—Crohn’s disease; UC—ulcerative colitis; SLUG—SNAIL family transcriptional repressor 2; IRS—immunoreactive score; nucl.—nuclear.

Statistical analysis of the results showed a significantly higher cytoplasmic IHC expression of SLUG in the stroma (stromal SLUG IRS) in IBD cases compared to the control group (*p* < 0.01, Mann–Whitney U test, Figure 5A). No statistically significant differences in stromal SLUG IRS by IHC were found in the group of patients with CD and UC compared to the control group (*p* > 0.05, Kruskal–Wallis test with Dunn post hoc test, Figure 5C). A comparative analysis of stromal SLUG IRS by IHC in connective tissue cells in individual sections of the intestine did not reveal any statistically significant differences (*p* > 0.05, Kruskal–Wallis test with Dunn post hoc test, Figure 5E).

#### 3.3.4. Analysis of Nuclear Expression of SLUG in the Connective Tissue of the Lamina Propria of the Mucosa and Submucosa

Statistical analysis of the results showed a significantly higher nuclear IHC expression of SLUG in connective tissue (stromal nuclear SLUG) in IBD cases compared to the control group (*p* < 0.05, Mann–Whitney U test, Figure 5B). No statistically significant differences in stromal nuclear SLUG expression by IHC were found in the group of patients with CD and UC compared to the control group (*p* > 0.05, Kruskal–Wallis test with Dunn post hoc test, Figure 5D). A comparative analysis of stromal nuclear SLUG expression by IHC in individual sections of the intestine did not reveal any statistically significant differences (*p* > 0.05, Kruskal–Wallis test with Dunn post hoc test, Figure 5F).

### 3.4. Analysis of SNAI2 mRNA Expression

**Figure 6:**
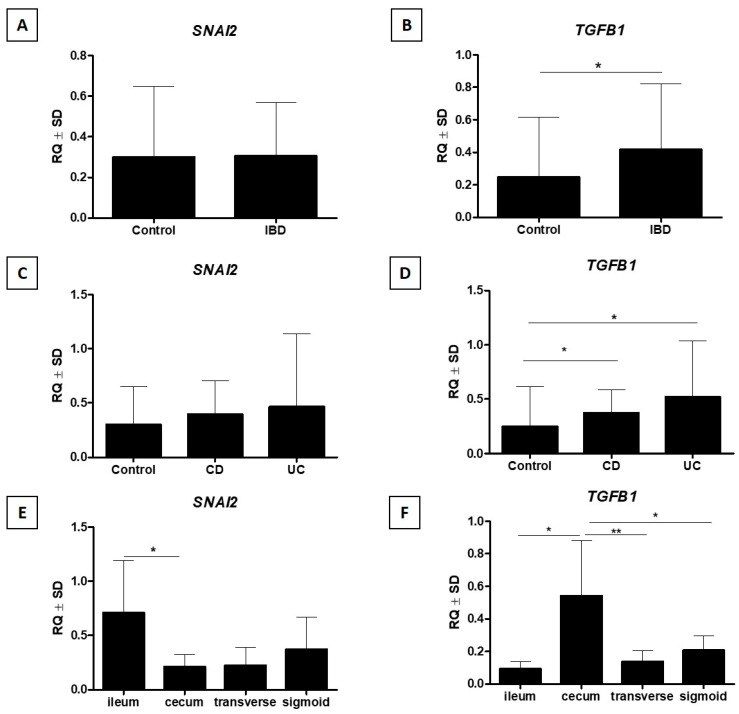
Statistical analysis of *SNAI2* and *TGF-β1* mRNA expression revealed that no significant differences in *SNAI2* mRNA expression were observed between the IBD and control groups (**A**), whereas *TGF-β1* mRNA expression was significantly higher in IBD compared with controls (**B**). Additionally, *SNAI2* mRNA expression showed no significant differences between the control, CD, and UC groups (**C**), while *TGF-β1* mRNA expression was higher in the CD and UC groups compared with controls (**D**). Furthermore, *SNAI2* mRNA expression was higher in the ileum compared with the cecum, while in the remaining large-intestine segments there was only a non-significant trend toward higher *SNAI2* expression in the ileum (**E**), and *TGF-β1* mRNA expression was higher in the cecum compared with the other intestinal segments examined (**F**) (respectively: Mann–Whitney U test (**A**,**B**), Kruskal–Wallis test with Dunn’s post hoc test (**C**–**F**), * *p* < 0.05, ** *p* < 0.01). IBD—inflammatory bowel disease; CD—Crohn’s disease; UC—ulcerative colitis; TGF-β—transforming growth factor β; SNAI2—SNAIL family transcriptional repressor 2.

Statistical analysis of *SNAI2* mRNA expression did not reveal any statistically significant differences in the expression of this marker in the group of patients with IBD compared to the control group (*p* > 0.05, Mann–Whitney U test, Figure 6A). Additionally, no significant differences in *SNAI2* expression were found between the groups of patients with CD, UC, and the control group (*p* > 0.05, Kruskal–Wallis test with Dunn post hoc test, Figure 6C). A comparative analysis of *SNAI2* expression in individual sections of the intestine showed only a trend towards higher expression of this marker in the ileum compared to other sections of the intestine (cecum, transverse colon, and sigmoid colon or rectum) (*p* > 0.05, Kruskal–Wallis test with Dunn post hoc test, Figure 6E). Detailed data are provided in Table 2.

### 3.5. Analysis of TGF-β Expression

Diverse cytoplasmic expression of TGF-β was demonstrated in epithelial cells and connective tissue of the lamina propria of the mucosa and submucosa of the intestine (stroma) (Figure 2E,F). Positive expression was demonstrated in all cases examined in the study group and in 7/10 and 8/10 controls, respectively, in the epithelium and connective tissue. Detailed data are provided in Table 1.

#### 3.5.1. Analysis of Cytoplasmic TGF-β Expression in Epithelial Cells

**Figure 7:**
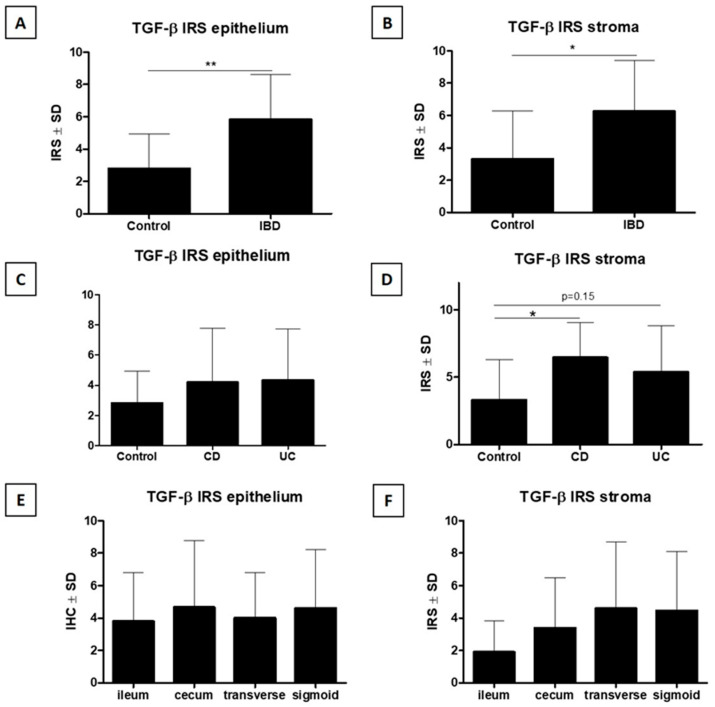
Significantly higher cytoplasmic TGF-β IRS by IHC in the epithelium (**A**) and submucosa (**B**) was observed in the IBD group compared with the control group. A more detailed analysis showed higher epithelial TGF-β IRS (**C**) in the CD and UC patient groups compared with the control group (statistically insignificant trend). Significantly higher submucosal TGF-β IRS was observed in the CD and UC groups compared with the control group (**D**). Additionally, epithelial TGF-β IRS did not differ significantly across the examined gastrointestinal segments (**E**), while in the submucosa a non-significant trend toward lower IRS was observed in the ileum compared with all large-intestine segments (**F**) (respectively: Mann–Whitney U test (**A**,**B**), Kruskal–Wallis test with Dunn’s post hoc test (**C**–**F**), * *p* < 0.05, ** *p* < 0.01). IBD—inflammatory bowel disease; CD—Crohn’s disease; UC—ulcerative colitis; TGF-β—transforming growth factor β; IRS—immunoreactive score.

Statistical analysis showed a significantly higher cytoplasmic TGF-β IHC expression in epithelial cells (epithelial TGF-β IRS) in IBD cases compared to the control group (*p* < 0.01, Mann–Whitney U test, Figure 7A). In addition, a statistically insignificant trend toward higher epithelial TGF-β IRS by IHC was demonstrated in the CD and UC patient groups compared to the control group (*p* > 0.05, Kruskal–Wallis test with Dunn post hoc test, Figure 7C). A comparative analysis of cytoplasmic TGF-β IHC expression in epithelial cells in individual sections of the intestine did not reveal any statistically significant differences between sections (*p* > 0.05, Kruskal–Wallis test with Dunn post hoc test, Figure 7E).

#### 3.5.2. Analysis of Cytoplasmic TGF-β Expression in Connective Tissue Cells of the Lamina Propria of the Mucosa and in the Submucosa

Statistical analysis of the results showed a significantly higher cytoplasmic TGF-β IHC expression in connective tissue cells (stromal TGF-β IRS) in IBD cases compared to the control group (*p* < 0.05, Mann–Whitney U test, Figure 7B). In addition, significantly higher IHC expression of stromal TGF-β IRS was demonstrated in the CD patient group and a trend towards higher IHC expression of stromal TGF-β IRS in the UC patient group compared to the control group (*p* = 0.054, Kruskal–Wallis test with Dunn post hoc test, Figure 7D). A comparative analysis of IHC expression of stromal TGF-β IRS in individual sections of the intestine showed a statistically insignificant trend toward higher IHC expression of stromal TGF-β IRS in the cecum, transverse colon, and sigmoid colon or rectum compared to the ileum (*p* > 0.05, Kruskal–Wallis test with Dunn post hoc test, Figure 7F).

### 3.6. Analysis of TGF-β1 mRNA Expression

Statistical analysis of *TGF-β1* mRNA expression showed significantly higher expression of this marker in IBD cases compared to the control group (*p* < 0.05, Mann–Whitney U test, Figure 6B). In addition, significantly higher *TGF-β1* mRNA expression was demonstrated in the group of patients with CD compared to the control group and compared to UC patients (*p* < 0.05, Kruskal–Wallis test with Dunn post hoc test, Figure 6D). A comparative analysis of *TGF-β1* expression in individual sections of the intestine showed a significantly higher expression of this marker in the cecum compared to other sections of the intestine (*p* < 0.01, Kruskal–Wallis test with Dunn post hoc test, Figure 6F). Detailed data are provided in Table 2.

### 3.7. Correlation Analysis Between mRNA Expression of Studied Genes

Correlation analysis showed a significant strong positive correlation between *POSTN* and *SNAI2*. Additionally, a significant moderate positive correlation between *POSTN* and *TGF-β1* and between *TGF-β1* and *SNAI2* were shown. Detailed data are presented in Table 4.

### 3.8. Correlation Analysis Between IHC Expression of the Studied Markers

Correlation analysis showed a statistically significant moderate positive correlation between periostin IRS and stromal nuclear SLUG expression. In addition, a statistically significant strong positive correlation between periostin IRS by IHC and stromal TGF-β IRS, as well as of stromal nuclear SLUG expression by IHC and stromal TGF-β IRS were shown. Detailed statistical data on the correlation analysis are presented in Table 5.

### 3.9. Correlation Analysis Between the Examined Markers and Clinical Data

Correlation analysis of periostin IRS by IHC in the connective tissue cells of the lamina propria of the mucosa and the submucosa with fecal calprotectin concentration showed a statistically significant strong negative correlation. Additionally, correlation analysis of stromal TGF-β IRS by IHC with fecal calprotectin concentration in stool showed a statistically significant moderate negative correlation. Furthermore, correlation analysis of periostin IRS by IHC in the connective tissue cells of the lamina propria of the mucosa and the submucosa with disease activity assessed according to the PCDAI scale showed a statistically significant moderate negative correlation. No statistically significant relationships were found with the other markers studied. Detailed statistical data on the correlation analysis are presented in Table 5 and Table 6.

### 3.10. Age and Gender

No associations between the examined markers and gene expression levels with patients’ age or gender were observed.

### 3.11. Post Hoc Power Analysis

A post hoc power analysis, performed using the classical analytical method based on the statistical distributions of the applied tests (t, F, or χ^2^), was conducted to estimate the achieved power of the study.

Assuming α = 0.05, a moderate effect size (Cohen’s d = 0.6), and the actual sample sizes for each comparison, the calculated power was 0.43 for periostin expression (ulcerative colitis vs. Crohn’s disease; Figure 3D) and 0.50 for nuclear SLUG expression in the submucosa (controls vs. IBD; Figure 5B). These values indicate limited power for detecting effects of moderate magnitude in these specific comparisons.

For all other subgroup analyses that reached statistical significance, the achieved power ranged from 0.70 to 0.99, indicating adequate sensitivity to detect effects of moderate size.

## 4. Discussion

In this study, we demonstrated that periostin and SLUG are significantly upregulated in intestinal tissue from pediatric patients with IBD, including both Crohn’s disease (CD) and ulcerative colitis (UC), compared to controls. Importantly, the expression of both markers showed a positive correlation with TGF-β and overlapping localization in the connective tissue, suggesting a coordinated role in epithelial–mesenchymal transition (EMT) and fibrosis of the chronically inflamed intestinal wall. To our knowledge, this is among the first studies to quantitatively and semi-quantitatively assess periostin at both the gene (RT-PCR) and protein (IHC) levels in pediatric IBD, addressing a critical gap in knowledge regarding the molecular mechanisms of intestinal fibrosis in this population [62,68,69,70]. These results confirm the involvement of periostin in IBD pathogenesis and indicate that periostin may actively contribute to tissue remodeling and EMT-associated processes in children.

Fibrosis represents one of the most clinically relevant consequences of chronic intestinal inflammation. In Crohn’s disease, persistent transmural inflammation promotes irreversible fibrotic remodeling, leading to strictures, obstruction, and penetrating complications, whereas in ulcerative colitis, fibrotic remodeling is usually limited to superficial mucosal layers [25,36,37,38,39,40,41,42]. More than half of CD patients eventually develop a stricturing or penetrating phenotype within two decades of diagnosis, and 70–80% require intestinal surgery [36,37]. Postoperative recurrence of fibrosis, particularly at the ileocecal anastomosis, is common and poses a major therapeutic challenge [38,39].

On a cellular level, chronic inflammation triggers EMT—a process in which epithelial cells lose polarity and intercellular adhesion and acquire mesenchymal properties such as motility and extracellular matrix production [43,44]. This process is regulated by transcription factors including SNAIL, SLUG, TWIST, ZEB1, and ZEB2, which downregulate epithelial markers like E-cadherin and induce mesenchymal proteins such as vimentin and N-cadherin [45]. EMT activation is driven by signaling pathways such as TGF-β, Wnt, and Notch and is strongly modulated by inflammatory cytokines and hypoxia [46].

Previous studies have shown marked overexpression of SNAIL and SLUG in damaged intestinal crypts of IBD patients, extending into mesothelial and subserosal fibroblasts, especially in fibrotic regions [47]. Scharl et al. demonstrated increased TGF-β1 expression and nuclear accumulation of SLUG in fibrotic intestinal areas, accompanied by β-catenin activation and fibroblast proliferation [48], while Schäfer et al. reported that TGF-β1 induces SLUG-dependent L1CAM upregulation, enhancing motility and apoptosis resistance [49]. These findings collectively underscore the role of inflammatory signaling in EMT-driven intestinal fibrosis, consistent with our observation of periostin–SLUG–TGF-β co-expression in pediatric IBD.

Although data on periostin expression in IBD are limited, previous studies provide important context. Kikuchi et al. reported high periostin expression in the lamina propria and pericryptal connective tissue of UC patients [69]. In experimental mouse models of colitis, *POSTN* knockout mice (*POSTN* −/−) exhibited reduced disease severity and fewer inflammatory cell infiltrates compared to wild-type controls after induction with DSS or TNBS, whereas administration of recombinant periostin restored severe inflammation, and neutralizing antibodies against periostin alleviated it [63,70]. These findings support the immunomodulatory role of periostin in regulating the recruitment and activity of inflammatory cells, including T lymphocytes, macrophages, and dendritic cells, in the intestinal mucosa. Consistent with these observations, we found higher expression of periostin and TGF-β in pediatric IBD tissues compared to controls, with a positive correlation between their mRNA and protein levels, suggesting that periostin may act in concert with TGF-β to promote chronic inflammatory and fibrotic processes [71].

**Figure 8:**
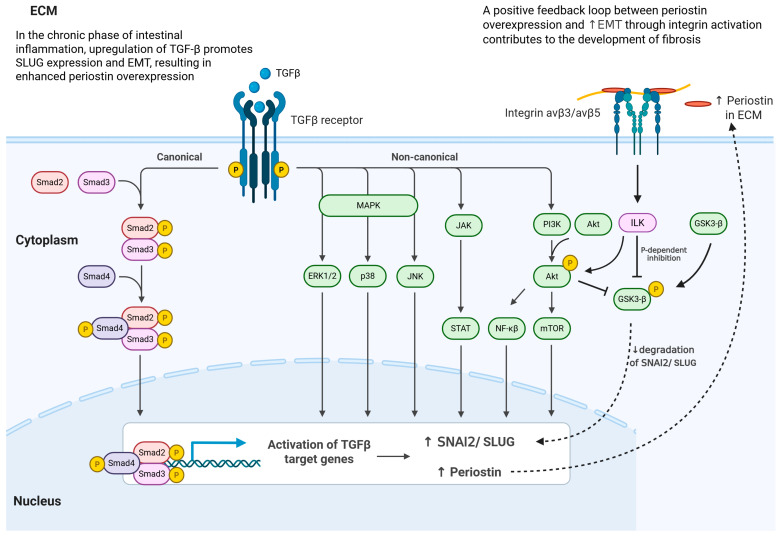
Schematic representation of TGF-β signaling pathways leading to EMT activation and periostin overexpression during intestinal inflammation. In response to intestinal inflammation, TGF-β expression is upregulated and activates both canonical and non-canonical signaling pathways through the TGF-β receptor. In the canonical pathway, phosphorylated Smad2/3 form a complex with Smad4 that translocates to the nucleus and induces target genes such as SNAI2/SLUG, promoting EMT and periostin production. Non-canonical signaling involves MAPK (ERK1/2, p38, JNK), JAK/STAT, and PI3K/Akt/mTOR cascades, further enhancing SNAI2/SLUG expression and preventing its degradation via GSK3-β inhibition. Periostin secreted into the ECM binds αvβ3/αvβ5 integrins, activating ILK and establishing a positive feedback loop that reinforces TGF-β signaling, promotes EMT, and contributes to tissue fibrosis. Created in BioRender. Grzegrzółka, J. (2026) https://BioRender.com/kkve26h (accessed on 4 January 2026).

A plausible mechanism is that TGF-β signaling induces periostin production, while periostin, through αvβ3/αvβ5 integrin and ILK activation, enhances TGF-β signaling and reinforces ECM deposition and EMT, forming a positive feedback loop that perpetuates fibrosis (as illustrated in Figure 8). This molecular interaction is well-documented in other tissues, further emphasizing periostin’s cross-organ profibrotic role [53,64,65,66].

A key strength of our study is the analysis of periostin directly in intestinal tissue rather than serum, which avoids potential confounding factors in the pediatric population. Notably, circulating periostin levels are highly influenced by age-dependent growth and bone metabolism, resulting in substantially higher plasma concentrations in children compared to adults, as well as by circadian variation, BMI, and smoking status [72,73,74,75,76,77,78]. This tissue-based approach allowed a more precise assessment of the local role of periostin in the intestinal wall, independent of systemic fluctuations. Our results also highlight the potential importance of periostin in the chronic phase of inflammation, when fibrotic remodeling predominates, suggesting that periostin may serve as a tissue biomarker for early fibrotic changes in pediatric IBD.

The relevance of periostin to fibrotic processes is further supported by its established role in fibrosis of multiple organs, including subepithelial bronchial fibrosis in asthma, idiopathic pulmonary fibrosis, myelofibrosis, systemic sclerosis, and post-ischemic myocardial fibrosis [60,61,68,79,80,81,82,83,84,85,86,87,88,89,90,91,92]. In these contexts, periostin not only reflects ongoing fibrogenesis but actively contributes to extracellular matrix (ECM) remodeling and EMT, processes that are mirrored in our observations of the intestinal wall in pediatric IBD. Furthermore, due to the specific role of the subepithelial connective tissue of the bronchial wall, circulating periostin concentrations in blood serum have been used as markers of asthma progression [73,77,93,94,95] or the advancement of idiopathic pulmonary fibrosis (particularly the monomeric form) [84,96], and pilot studies suggest its potential utility as a biomarker for airway epithelial inflammation, including allergic rhinitis [97].

SLUG, a transcription factor and recognized marker of EMT [98], was also significantly upregulated in our IBD cohort, with localization overlapping periostin and TGF-β. This supports the notion that chronic intestinal inflammation induces EMT in epithelial and connective tissue cells, with periostin potentially facilitating this process, as seen in other fibrotic and cancerous tissues [99,100]. A molecular mechanism potentially driving excessive fibrosis is presented in Figure 8.

Our study also examined correlations between markers and clinical parameters. We observed a significant negative correlation between periostin expression and fecal calprotectin, as well as a moderate negative correlation between of TGF-β expression and fecal calprotectin, suggesting that periostin may be particularly elevated during phases of intestinal repair and regeneration when active neutrophilic inflammation is reduced. In addition in CD patients, periostin expression correlated negatively with PCDAI scores, further supporting its association with chronic, profibrotic stages rather than acute inflammation [22,62]. In experimental acute kidney injury models, periostin has been shown to modulate macrophage behavior, promoting proliferation and polarization toward an M2 phenotype [101]. We propose that a similar mechanism could explain the observed reduction of mononuclear cell infiltrates, the main source of calprotectin overproduction in the intestinal wall.

Interestingly, no correlation was observed between periostin expression and PUCAI in UC, indicating potential differences in fibrotic processes and marker expression between IBD subtypes [102,103]. These findings, along with the higher periostin levels in CD compared to UC, underscore the molecular and clinical heterogeneity of pediatric IBD.

Beyond its diagnostic potential, periostin may have therapeutic relevance. Elevated tissue and serum periostin levels have been linked to type 2 inflammatory responses mediated by IL-4 and IL-13 [104], and interventions targeting periostin modulate inflammation in experimental models. Consequently, periostin may serve as a biomarker for fibrotic progression, a predictor of response to anti-inflammatory therapy, and a potential target for novel therapeutics in pediatric IBD [105]. Environmental and lifestyle factors may further influence periostin, TGF-β, and SLUG expression, highlighting the need for future studies to explore these modulatory effects. For instance, periostin is upregulated by Th2 cytokines (IL-4, IL-13), which can be affected by allergen exposure, physical activity, smoking, and diet [68,106,107]. These findings suggest that environmental and lifestyle exposures could contribute to interindividual variability in marker expression and the progression of chronic inflammation and fibrosis in IBD.

Chronic inflammatory conditions such as IBD are well-established risk factors for colitis-associated colorectal cancer, as persistent inflammation and repeated cycles of injury and repair can promote epithelial DNA damage, dysplasia, and eventual neoplastic transformation over time. Periostin may contribute to tumorigenesis by interacting with integrins and promoting a microenvironment that supports cell proliferation and survival, while SLUG and TGF-β are known to facilitate EMT and invasiveness, processes associated with cancer development [108,109,110,111].

Despite these promising results, our study has limitations. The primary drawback is the relatively small, single-center cohort, which may limit generalizability. Although the final sample size was slightly smaller than planned, statistically significant differences were observed for the main comparisons. Post hoc power analysis indicated limited sensitivity for periostin and nuclear SLUG expression, suggesting caution in interpreting these results. Nevertheless, large effect sizes support the biological relevance of the findings, and other significant analyses had adequate power (0.70–0.99). Replication in larger, multicenter cohorts with longitudinal follow-up is warranted to confirm these observations and further elucidate periostin’s role in chronic inflammation, EMT, and fibrogenesis in IBD.

In conclusion, our study suggests that periostin, in association with SLUG and TGF-β, is upregulated in pediatric IBD and may play a role in EMT and intestinal fibrosis. These findings contribute to the limited understanding of fibrotic mechanisms in children with IBD, highlight potential pathways linking chronic inflammation to tissue remodeling, and support further investigation of periostin as a possible biomarker or therapeutic target.

## 5. Conclusions

1. Higher expression of periostin, TGF-β, and SLUG in pIBD cases compared to controls suggests a role for all these markers in the pathogenesis of IBD.
2. The difference in the level of periostin expression between CD and UC may suggest a different etiology of these two IBD diseases.
3. The observed associations between periostin, TGF-β, and SLUG expression may indicate a potential link between periostin and molecular mechanisms underlying chronic inflammation and fibrosis in the intestinal wall.
4. Negative correlations between periostin expression and calprotectin and PUCAI/PCDAI concentrations may suggest that periostin exacerbates pro-fibrotic processes in the chronic phase of IBD, which may be an interesting aspect for further research.

## Figures and Tables

**Table 1:** Clinical characteristics of patients and immunohistochemical expression of the examined markers assessed by IHC. *p*-values indicate the significance of differences between groups (IBD vs. control) as determined by the Mann–Whitney U test.

Parameters	IHC		
	IBD	Control	*p*-Value
**Number of cases**	33	10	
CU	11		
CD	22		
Age (mean ± SD)	14.5 ± 5.6	13.0 ± 6.6	*p* = 0.11
**Gender**			
Male	13	5	
Female	20	5	
Concentration of calprotectin (ug/g) (mean ± SD)	1591 ± 3163	87.6 ± 250	*p* < 0.0001
PUCAI (mean ± SD)	27.9 ± 16.5		
PCDAI (mean ± SD)	25.7 ± 18.5		
**Section of intestine**			
Ileum	22		
Cecum	26		
Transverse	19		
sigmoid/rectum	25		
**Expression of markers:**			
**periostin (mean ± SD)**			
periostin IRS submucosal (mean ± SD)	3.6 ± 2.6	2.1 ± 1.8	*p* < 0.05
Positive	33	9	
Negative	0	1	
**SLUG (mean ± SD)**			
SLUG nuclear epithelial (mean ± SD)	2.2 ± 1.7	0.6 ± 0.5	*p* < 0.05
Positive	22	6	
Negative	11	4	
SLUG IRS epithelial (mean ± SD)	8.4 ± 3.2	4.9 ± 3.6	*p* < 0.01
Positive	32	8	
Negative	1	2	
SLUG nuclear submucosal (mean ± SD)	2.7 ± 1.3	1.7 ± 1.2	*p* < 0.05
Positive	30	8	
Negative	3	2	
SLUG IRS submucosal (mean ± SD)	4.0 ± 3.5	1.0 ± 0.9	*p* < 0.01
Positive	25	6	
Negative	8	4	
**TGF-β (mean ± SD)**			
TGF-β IRS epithelial (mean ± SD)	5.9 ± 2.8	2.8 ± 2.2	*p* < 0.01
Positive	33	7	
Negative	0	3	
TGF-β IRS submucosal (mean ± SD)	6.2 ± 3.1	3.3 ± 3.0	*p* < 0.05
Positive	33	8	
Negative	0	2	

Note: Bold font indicates column and section headers.

**Table 2:** Clinical characteristics of patients and expression of the examined genes assessed by quantitative PCR (qPCR). *p*-values indicate the significance of differences between groups (IBD vs. control) as determined by the Mann–Whitney U test.

Parameters	qPCR		
	IBD	Control	*p*-Value
**Number of cases**	22	6	
CU	11		
CD	11		
Age (mean ± SD)	13.2 ± 1.9	12.2 ± 1.9	*p* = 0.25
**Gender**			
Male	7	3	
Female	15	3	
Concentration of calprotectin (ug/g) (mean ± SD)	3935 ± 4891	328.4 ± 430.5	*p* < 0.05
PUCAI (mean ± SD)	25.7 ± 16.6		
PCDAI (mean ± SD)	30.0 ± 7.0		
**Section of intestine**			
Ileum	2		
Cecum	8		
Transverse	5		
sigmoid/rectum	7		
**Expression of markers:**			
*POSTN* (mean ± SD)	1.0 ± 1.5	0.3 ± 0.4	*p* < 0.05
*SNAI2* (mean ± SD)	0.3 ± 0.3	0.3 ± 0.4	*p* = 0.71
*TGF-β1* (mean ± SD)	0.4 ± 0.4	0.3 ± 0.4	*p* < 0.05

Note: Bold font indicates column and section headers.

**Table 3:** Evaluation of immunohistochemical staining according to Remmele and Stegner.

Points	Intensity of Staining	Percentage of Positive Cells
0	negative staining	0%
1	low-intensity staining	<10%
2	moderate staining	10–50%
3	intense staining	51–80%
4	-	81–100%

**Table 4:** Spearman correlations between *POSTN*, *SNAI2*, and *TGF-β1* mRNA expression levels.

	*POSTN*	*SNAI2*	*TGF-β1*
***POSTN***	-	0.645 *	0.448 *
***SNAI2***	0.645 *	-	0.445 *
***TGF-β1***	0.448 *	0.445 *	-

* *p* < 0.05.

**Table 5:** Spearman correlations between periostin IRS, stromal nuclear SLUG, stromal TGF-β IRS protein expression levels, and fecal calprotectin concentration.

	Periostin IRS	Stromal Nuclear SLUG	Stromal TGF-β IRS	Calprotectin
Periostin IRS	-	0.382 *	0.598 ***	−0.590 ***
Stromal nuclear SLUG	0.382 *	-	0.555 ***	0.207
Stromal TGF-β IRS	0.598 ***	0.555 ***	-	−0.438 *
Calprotectin	−0.590 ***	0.207	−0.438 *	-

* *p* < 0.05; *** *p* < 0.001.

**Table 6:** Spearman correlations between periostin IRS, stromal nuclear SLUG, and stromal TGF-β IRS protein expression levels and PCDAI and PUCAI scores.

	PCDAI	PUCAI
Periostin IRS	−0.762 *	−0.377
Stromal TGF-β IRS	−0.333	0.822 *
Stromal nuclear SLUG	0.866	−0.156

* *p* < 0.05.

## Data Availability

The data presented in this study are available on request from the corresponding author. The data are not publicly available due to privacy issues.

## Abbreviations

The following abbreviations are used in this manuscript:

pIBD	Pediatric inflammatory bowel disease
CD	Crohn’s disease
UC	Ulcerative colitis
IBD-U	IBD-unclassified
IBD	Inflammatory bowel disease
VEO-IBD	very early-onset IBD
PCDAI	Pediatric Crohn’s Disease Activity Index
PUCAI	Pediatric Ulcerative Colitis Activity Index
TGF-β	transforming growth factor β
SLUG	SNAIL family transcriptional repressor 2
TNF-α	tumor necrosis factor-α
EMT	epithelial–mesenchymal transition
SNAIL	SNAIL family transcriptional repressor 1
TWIST	twist family bHLH transcription factor 1
ZEB1	Zinc finger E-box-binding homeobox 1
ZEB2	Zinc finger E-box-binding homeobox 2
Wnt	Wingless/Int-1
IL-6	interleukin 6
IL-1β	interleukin 1β
IRS	immunoreactive score
ECM	extracellular matrix
IHC	Immunohistochemical reactions
DSS	dextran sulftate sodium
TNBS	trinitrobenzene sulfonic acid
CCR5	C-C chemokine receptor 5
BMI	body mass index
CRC	colorectal cancer
IPF	idiopathic pulmonary fibrosis
PCR	polymerase chain reaction
JAK	Janus-Activated Kinases
STAT	Signal Transducer and Activator of Transcription
MAPK	mitogen-activated protein kinases
mTOR	mechanistic Target of Rapamycin
GSK3-β	Glycogen Synthase Kinase 3 beta
ILK	Integrin-Linked Kinase
